# Supplementary material for: Development and Characterization of Two Wheat–Rye Introgression Lines with Resistance to Stripe Rust and Powdery Mildew
Source: Int J Mol Sci. 2024 Oct 30;25(21):11677. doi: 10.3390/ijms252111677 (PMC11546993; doi:10.3390/ijms252111677)
Supplement: Supplementary file 1 [file ijms-25-11677-s001.zip › ijms-3192175-supplementary.pdf]

Table S1 The specific molecular marker information.

Marker name	Primer sequence (5'-3')
TNAC1142F	CAGCATCCATAACCAGGATGT
TNAC1142R	GCCTACGAGTACATGGTCGAG
TNAC1144F	CCATGTTCAAGAGGATCAACG
TNAC1144R	GTGGGTATTCCCAACTCCTTG
SW17412F	GATGGCCACGATAGAGGAGA
SW17412R	TAAACACTTGCGCTTACCGA
SW17933F	ATCAGCATCCACACTCACCA
SW17933R	TGCAGTCGCATTAGCTTCAG
SW19466F	GCCATCAGTGGAGAAGATCAG
SW19466R	GCGTGATGGAACATGACAAC
SW24019F	TGGTAGGTTTGGCTACGAGG
SW24019R	CGAGAGACTGAGCCGACATA
SW131261F	TTGTGGCATTCAATCAGGAA
SW131261R	TACGAACCTGCAGTGGATTG
